# Epidemioclinical profile and psychological experience of women consulting for primary infertility at the university hospital of angre about 160 cases

**DOI:** 10.3389/frph.2025.1597911

**Published:** 2026-01-23

**Authors:** Gbary-Lagaud Eléonore, Houphouet-Mwandji Carine, Kouakou-Kouraogo Ramata, Effoh Denis, Adjoby Roland

**Affiliations:** Department of Mother and Child, Félix Houphouët Boigny University, Abidjan, Côte d'Ivoire

**Keywords:** infertility, upper genital infection, cycle disorder, fibroid, anxiety, sexual disorders, access to care

## Abstract

**Background:**

Infertility is a real public health problem today with clinical and psychological aspects.

**Objective:**

To improve the care and monitoring of women with primary infertility at the Angré University Hospital Center.

**Materials and method:**

This was a cross-sectional and descriptive study over a period from January 1, 2021 to December 31, 2023. It concerned all women who came to consult for an inability to procreate dating back more than one year without using a contraceptive method, having regular and complete intercourse and having never become pregnant.

**Results:**

Out of 7,348 gynecological consultations during the study period, 595 or 8% were related to infertility. The average age of the patients was 34.46 years (±5.9). Women with a higher education level were 56.3%. They were obese in 29.3% of cases. Genital infection (53.3%) was the main medical history in 53.3% of cases while the surgical history was dominated by myomectomy (44.7%) and appendectomy (38.3%). Among the causes of infertility, there was tubal obstruction (36.5%) followed by cycle irregularity and fibroids at 29% each. The main psychological disorders observed were anxiety (81.9%), sexual disturbances (56.3%) and stress (71.9%).

**Conclusion:**

Primary infertility is becoming increasingly common in our context and affects increasingly younger women. It would be wise to now include its medical and psychological management in an inclusive health program for all through universal health coverage.

## 1 Introduction

The World Health Organisation (WHO) defines infertility as the absence of conception after at least 12 months of regular and complete unprotected sexual intercourse without a contraceptive method (1). Infertility is responsible for a number of social issues: the physical and mental health of women and, ultimately, the survival of the human race (2, 3). Infertility is currently a public health problem, both because of its increasing frequency and because of the social issues involved: the physical and mental health of women, and ultimately the survival of the human race (3, 4). It is estimated that the worldwide prevalence of infertility is 46.25% and that of primary infertility 51.5% (5). In France, 15%–25% of couples consult a doctor each year for infertility (6). In Cameroon, infertility accounts for 20%–30% of gynaecological consultations (7). In Côte d'Ivoire, the prevalence of female infertility was 41% in 2016 (5). Its discovery is always a psychological trauma for the couple and the consequences can be multiple: depression, sexual disorders, extramarital sexuality leading to divorce and finally an identity crisis (5, 8, 9) especially in cases of primary infertility.

We took an interest in this and conducted a study at the Angré University Hospital to improve the management and follow-up of women with primary infertility.

## 2 Materials and methods

This was a cross-sectional and descriptive study over a period from January 1, 2021 to December 31, 2023, i.e., 36 months. The study population included all women who came for consultation for infertility. In other words women who have come to the clinic because they have been unable to have children for more than a year without using a contraceptive method and who have regular intercourse or 595 patients. We then removed patients with secondary infertility, or 435 patients. This is therefore a random sample.The inclusion criteria were as follows: women who came for consultation for primary infertility and who had completed the entire assessment of the initial consultation for infertility. Women with incomplete and/or unusable records and those not living with a partner were not included. Our sample size (*N* = 160) was determined by the Schwartz formula:

-*N* = [t²*p*(1- p)]/c^2^
-With: t = 1.96 (for a 95% confidence level)
P = proportion of the population coming for consultation, i.e., 9% (10);
C = the confidence level expressed, i.e., a 95% threshold.

To obtain results with a maximum error of 5.00%, the minimum sample size *n* is:

*N* = [1.96^2^*0.9*(1 − 0.9)]/0.05^2^
*N* = 138.29.

The variables studied were quantitative [age, body mass index, anti-Mullerian hormone (AMH), spermogram] on the one hand and qualitative (professional category, level of education, medical, surgical, gynecological history, psychosocial repercussions, post-coital Hühnner test) on the other hand. Statistical analysis of the data was performed using Epi Info 7 software.

All patients participated in the study after free and informed consent. Authorization for the study was obtained in advance from the hospital's medical and scientific director (Issue no. 20 of March 07, 2023). Anonymity and respect for data confidentiality were strictly observed.The survey was carried out by a doctor from the obstetrics and gynecology department using a standardized survey form. It should be noted that a few operational definitions are necessary. We have defined tubal obstruction as any abnormality in the passage of radio-opaque fluid through the fallopian tubes, as determined by hysterosalpingography. Anonymity and data confidentiality were strictly respected. Anxiety has been defined in our study as a state of painful worry, caused by uncertainty, expectation; anxiety, feeling of insecurity, tiredness, stress, headaches. Menstrual irregularity was defined as any abnormality in the menstrual cycle (shortening, lengthening, metrorrhagia, menorrhagia, intense dysmenorrhoea affecting daily activity).

## 3 Results

### 3.1 Frequency

**Figure 1:**
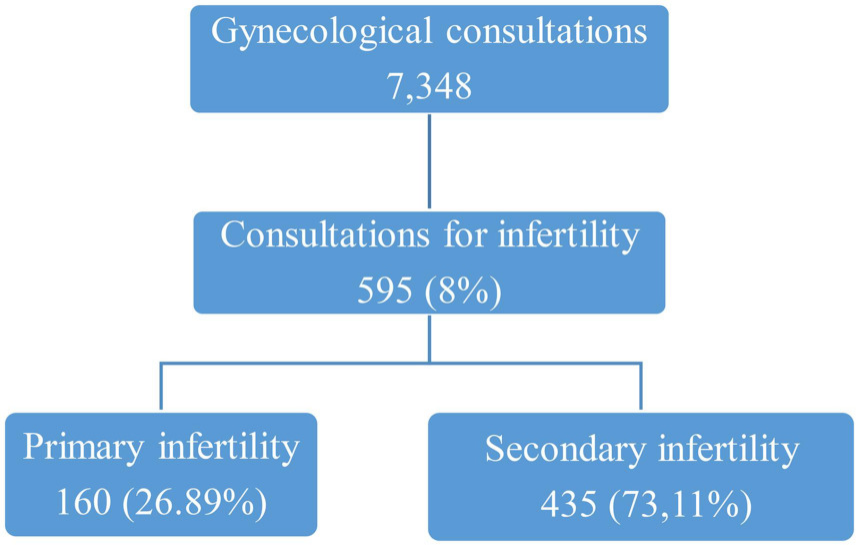
Flowchart for the frequency of primary infertility.

During the study period, we recorded 7,348 gynecological consultations. Primary infertility accounted for 2.18% of total consultations. The results are presented in the following flow diagram (Figure 1).

### 3.2 Socio-epidemiological characteristics

We were interested in the patients’ age, body mass index (BMI), education level, and socio-professional category. The mean age for primary infertility consultation was 34.6 years (± 5.9) with extremes of 25 years and 50 years (Table 1).

**Table 1:** Distribution of patients according to socio-epidemiological data for women consulting for primary infertility at the university hospital of angre from January 1, 2021 to December 31, 2023.

Socio-epidemiological characteristics	Frequency (*n*)	Percentage (%)
Age [31–35]	54	33.8
BMI > 30 (obesity)	47	29.3
Higher education level	90	56.25
Managers and employees	70	43.75

### 3.3 Clinical features

#### 3.3.1 Medical history

Medical history was selected based on its link to infertility. It should be noted that 100 women, or 62.2% of cases, had no specific medical history.

**Figure 2:**
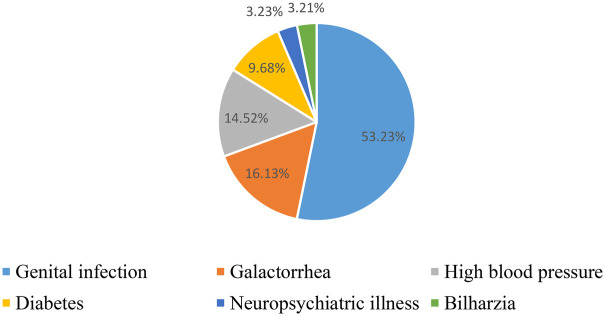
Distribution of women according to medical history related to infertility (*N* = 60) for women consulting for primary infertility at the university hospital of angre from January 1, 2021 to December 31, 2023.

Those with a medical history of genital infection were the most represented (Figure 2).

#### 3.3.2 Surgical history

Surgical history was unusual in 47 women in our study. In 113 cases, or 70.62%, there was no specific surgical history.

**Figure 3:**
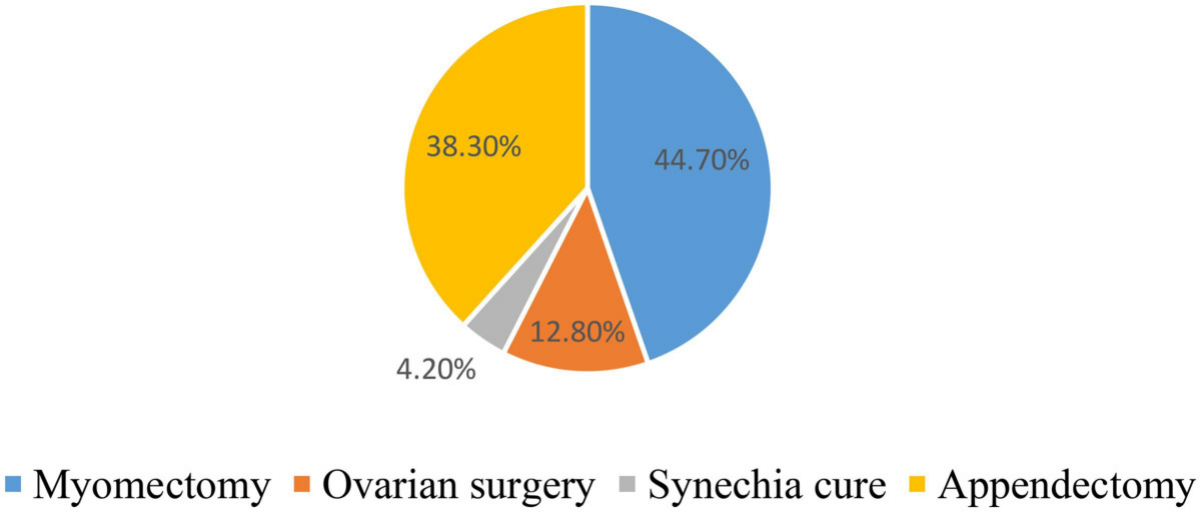
Distribution of women according to surgical history (*N* = 47) for women consulting for primary infertility at the university hospital of angre from January 1, 2021 to December 31, 2023.

Myomectomy was the most common surgical procedure performed (Figure 3).

#### 3.3.3 Gynecological history

**Figure 4:**
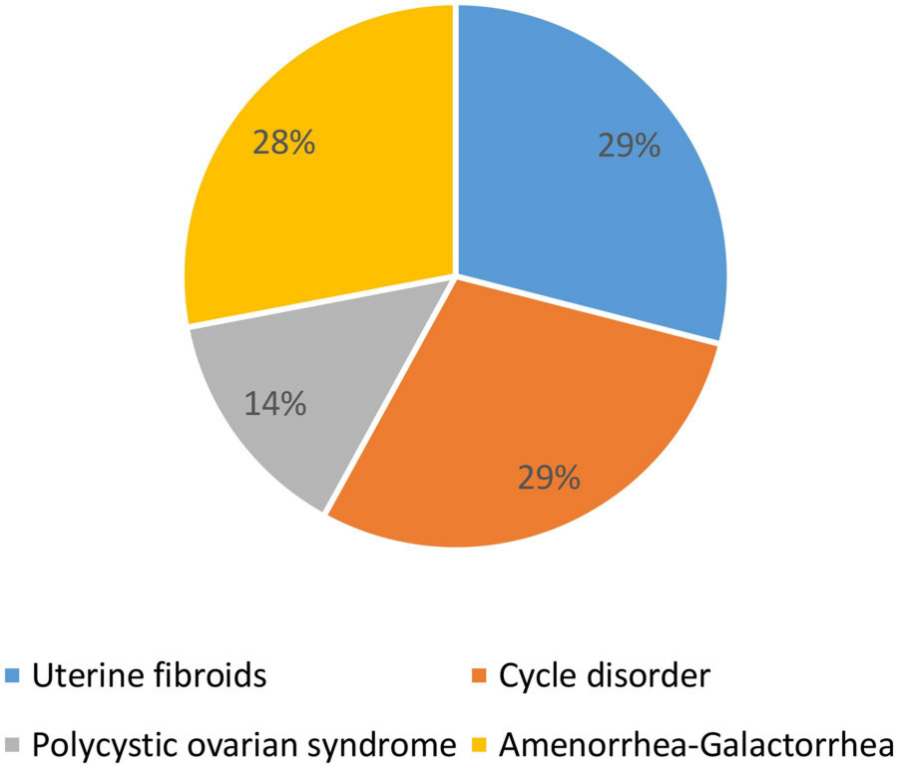
Distribution of women according to gynecological history (*N* = 93) for women consulting for primary infertility at the university hospital of angre from January 1, 2021 to December 31, 2023.

Cyclic irregularities and fibroids each accounted for 29% of cases. It should be noted that 93 women had specific gynecological histories, or 58.12% (Figure 4).

### 3.4 Paraclinical characteristics

#### 3.4.1 Biological data

For each patient, a biological work-up was carried out to search for a cause of primary infertility. We used AMH, post-coital Hühner test and spermogram data. A total of 59 spouses agreed to perform the requested spermogram, 54.2% of whom had a normal spermogram. The most frequently performed hormonal test was AMH in 24.1% (*N* = 34). The Hühner post-coital test, performed by 43 women between 6 and 8 h after sexual intercourse in the pre-ovulatory phase (Table 2).

**Table 2:** Breakdown by hormone status for women consulting for primary infertility at the university hospital of angre from January 1, 2021 to December 31, 2023.

Biological examination	Frequency (n)	Percentage (%)
AMH low	20	58
Post-coital Hühner test negative	13	30.2
Normal spermogram	32	54.2

#### 3.4.2 Imaging data

##### 3.4.2.1 Hysterosalpingography

**Figure 5:**
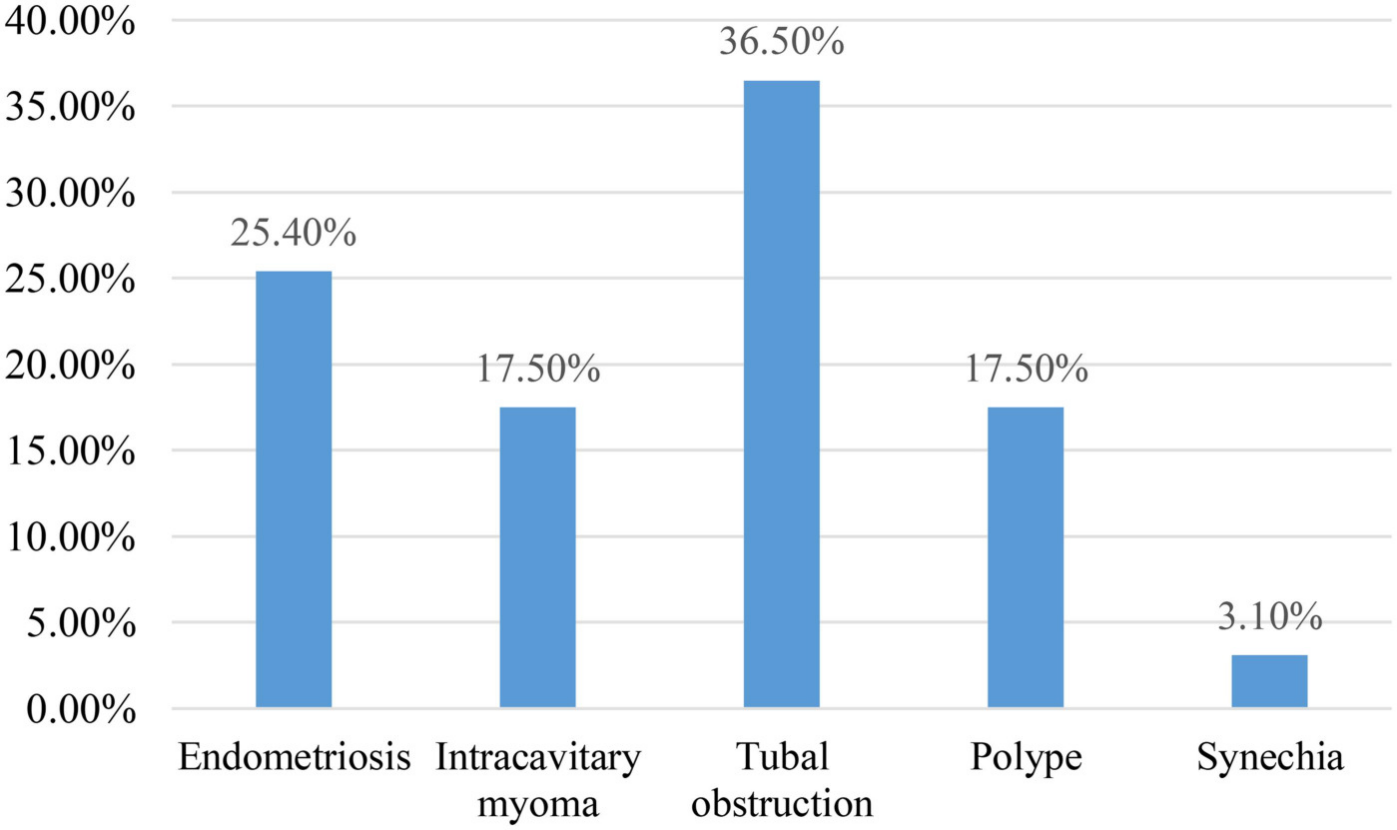
Distribution of women according to hysterosalpingography data for women consulting for primary infertility at the university hospital of angre from January 1, 2021 to December 31, 2023.

Patients were divided according to hysterosalpingography findings (*N* = 63), with tubal obstruction the most common cause at 36.5% (confidence interval 95% between 16 and 31) (Figure 5).

##### 3.4.2.2 Pelvic ultrasound

**Figure 6:**
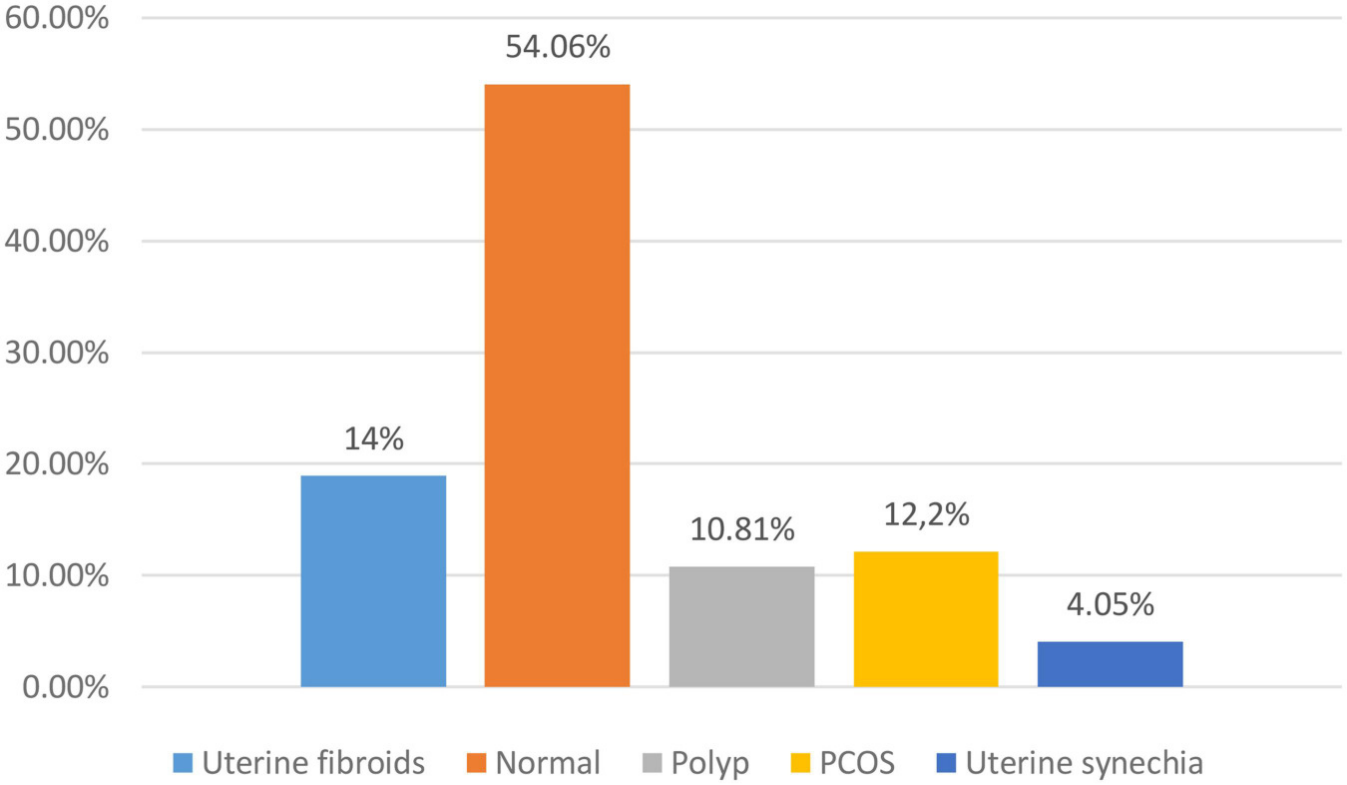
Distribution of women according to pelvic ultrasound data (*N* = 74) for women consulting for primary infertility at the university hospital of angre from January 1, 2021 to December 31, 2023.

Of the 74 women who underwent pelvic ultrasound, 54.1% had normal results. The anomalies encountered were mainly fibroids (14%) and polycystic ovarian syndrome (PCOS 12.2%) (Figure 6).

**Figure 7:**
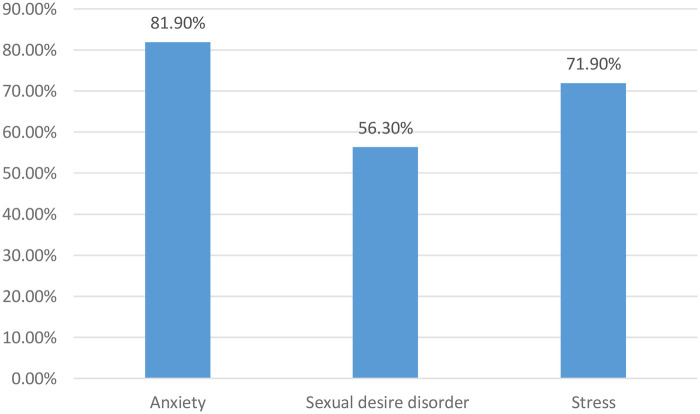
Distribution of women according to psychological disorders observed for women consulting for primary infertility at the university hospital of angre from January 1, 2021 to December 31, 2023.

### 3.5 Psychological aspect of infertile women

#### 3.5.1 Psychological disorders observed

The main disorders observed were anxiety (81.9% confidence interval 95% between 120 and 140 (Table 3).

**Table 3:** Distribution of women according to signs of psychosocial distress experienced.

Psychosocial disorder	Frequency (*n*)	Percentage (%)
Are you satisfied with the support you receive from friends and family?		
Yes	38	23.7
No	122	76.3
Do you feel angry about this situation?		
Yes	63	39.4
No	97	60.6
Are you experiencing a drop in self-confidence?		
Yes	44	27.5
No	116	72.5
Do you benefit from your spouse's support?		
Yes	82	51.2
No	78	48.8
Do you feel you're not a real woman?		
Yes	73	45.6
No	87	54.4
Have you been a victim of discrimination?		
Yes	38	23.8
No	122	76.2
Have you had a threat of marital separation because of this situation?		
Yes	36	22.5
No	124	77.5
Do you hope to have a child?		
Yes	122	76.2
No	38	23.8
Would you have liked psychological support?		
Yes	42	26.3
No	118	73.7
Have you used non-medical practices?		
Yes	108	67.5
No	52	32.5
Do you find it difficult to carry out your professional activities?		
Yes	100	62.5
No	60	37.5

#### 3.5.2 Psychosocial distress

The patients were divided according to the psycho-social distress experienced, on the one hand in relation to their loved ones, and on the other in relation to their spouse. Of the patients surveyed, 76.3% were dissatisfied with the support they received from their loved ones; 39.4% were angry about the situation. Those with reduced self-confidence accounted for 27.5%. 51.2% of patients received support from their spouses; those who had been discriminated against accounted for 23.8%, while 22.5% were threatened with separation.

Patients who no longer hoped to have a child represented 23.8%; those who had resorted to non-medical practices were 67.5%; difficulties in carrying out professional activities were encountered by 62.5% of them.

## 4 Discussion

### 4.1 Frequency

Our prevalence of primary infertility is higher than that of other authors. In a cross-sectional study of 1,067 women, Kazemijaliseh found a prevalence of primary infertility of 17.3% (11). This disparity can be explained by the recruitment method and the size of the different samples, but also by the time. In fact, the prevalence of infertility tends to increase over time (12) in relation to several factors: late pregnancies in couples, alterations in sperm quality due to habits such as smoking and alcohol, and changes in sexual behaviour (13). There is a disparity in the prevalence of infertility between developed and developing countries: 3.5%–16.7% compared with 6.9%–9.3%, with a median overall prevalence estimated at 9% (10). This difference is also due to the greater access to healthcare (14) in the Angré area, an upmarket district of Abidjan where the population has good purchasing power.

### 4.2 Socio-demographic characteristics of patients

The average age in our study is similar to that of Maï Abdessalem (10) who found an average age of 33 years. Our results are also similar to those found by Meka et al. (15) in their study of the knowledge, attitudes and practices of women wanting children in relation to infertility in Yaoundé, who found an average age of 31.07 years (16). This could be explained by the fact that women are increasingly studying and are primarily interested in their professional lives, hence the increase in the number of women over 30 coming to consult for primary infertility, as in our sample. A woman's age is one of the most important prognostic factors in infertility (17).

The relationship between weight and infertility was demonstrated as long ago as 1998 in the study by Green et al. (18), in which they found that overweight women were 4–7 times more likely to suffer from infertility and ovulation disorders. In our study 31.3% were overweight and 29.3% obese. Our results are similar to those of Maï Abdessalem (19) who found 32.5% overweight and 12.3% obese. Obesity can have a negative impact on fertility, it can lead to menstrual irregularities, it can also increase the risk of miscarriage and it also has a negative effect on the results of medically assisted reproduction techniques as demonstrated by Mohan and Sultana in a study carried out in 2001 (20).

Higher levels of education and socio-professional status are factors that delay the desire to have children. This observation has been made by authors in other countries (15). We can therefore agree that higher education has a significant impact on slowing down women's desire to have children. Women are increasingly giving priority to a stable career before thinking about conceiving. More and more women in our society are postponing their marriages in search of an advanced education that will enable them to enjoy a better social life, unlike in the old days when our parents and grandparents in Africa married early.

### 4.3 Clinical features

In many studies, genital infection is one of the main causes of infertility, with the complications of pelvic inflammation that it may cause (21). Kalume et al. (22), in a study of the clinical and aetiological profile of female infertility, found a high involvement of genital infection with pelvic tubal involvement in 67.6%. These results are similar to our own.

A history of pelvic surgery was associated with female infertility, as found in our study. In France, Ohannessian et al. (23) found that a history of appendectomy increased the risk of infertility by a factor of 5, a history of proven salpingitis by a factor of 32 and sexually transmitted infections by a factor of 8.

### 4.4 Biological characteristics

#### 4.4.1 Biological data

The post-coital Hühner test was carried out in the pre-ovulatory phase 6–8 h after intercourse. We found 30.2% negative and 21% positive tests. Our results are similar to those found in the literature. Afoutou (24) found 17% positive tests. Benmadjate (25) found 64% negative and 24% positive post-coital tests in his study. The physical and chemical properties of cervical mucus determine whether spermatozoa penetrate the cervix and reach the fertilisation site. Cervical mucus can therefore act either as an agent or as a physical and chemical barrier to conception. Cervical mucus can be used as a study medium where its changing properties can be exploited either to improve fertility or as a means of contraception.

In terms of hormonal data, AMH is one of the best indicators of a woman's fertility. This hormone provides information about ovarian reserve and therefore the woman's ability to procreate. Low levels indicate low ovarian reserve. This was found in 58% of our patients.

The spermogram is a fundamental test for assessing male fertility. The sampling conditions must be optimal to avoid altering the sperm and thus biasing the results (26). With industrialisation and changes in lifestyle, sperm abnormalities are more frequent, particularly in relation to slow, passive intoxication by cadnium, which is present in cigarettes, water and food (27).

#### 4.4.2 Data from hysterosalpingography

Bashiru et al. (28) in a study on the contribution of hysterosalpingography in the aetiological diagnosis of female infertility found a preponderance of mechanical causes, i.e., 33.5% of uterine fibroids, these results are close to those we found, i.e., 29% of fibroids. We also found 29% of menstrual irregularity and 26.9% of amenorrhoea-galactorrhoea syndrome. Coulibaly A et al. (29) in their study on the role of uterine fibroids in patients consulting for infertility found that 13.56% of cases of infertility were associated with the presence of uterine fibroids and nulligravida were the most represented with 38.76%.

The causes of infertility that we found did not differ from those described in the literature. These causes were dominated by tubal obstruction, endometriosis, myomas, polyps and sometimes synechiae. Tubal causes top the list as the aetiology of female infertility in many studies (30, 31). Because of their discreet symptoms, upper genital infections are often under-diagnosed. As a result, they are poorly treated or not treated at all, leading to irreversible damage to the tubal endothelium.

In many ways, endometriosis is a cause of infertility (32). The problem is that it is often unrecognised and difficult and late to diagnose. And even when the diagnosis is made, the question arises as to how to manage it, for which there is not always a consensus.

#### 4.4.3 Pelvic ultrasound data

The anomalies encountered were mainly fibroids (14%) and PCOS (12.2%). There is a harmful effect of uterine fibroids on fertility, particularly for those FIGO 0, 1, 2 which deform the uterine cavity (33). This is not clearly demonstrated but rather suggested. Polycystic ovary syndrome (PCOS) is a predominant cause of infertility and a common gynoendocrine disorder affecting 7%–15% of women of childbearing age (34). A healthy lifestyle with regular physical activity and weight loss help to reduce the dysovulation observed in PCOS. Treatment is based on clomiphene citrate combined to a greater or lesser extent with metformin (34). However, myoinositol is emerging as a therapeutic alternative (35).

### 4.5 Psychosocial aspects of couple infertility

Infertility is linked to a number of psychological disorders, including anxiety, depression and stress, which have a psychosocial impact on women with infertility (5, 36). Our data are in line with the literature. Nana (16) found that 84.6% of spouses were anxious and 84.61% were stressed. According to Élodie Girard et al. (37), more than 40% of infertile women present psychological disorders such as anxiety or depression, at levels equivalent to those of women suffering from chronic illnesses such as cancer, heart disease or HIV. In the African context, this stress is exacerbated by the views of the extended family and society, which is pro-natalist and has little sympathy for a childless couple. This psychological state has an impact on the performance of professional activities. In his study, Braverman also found a profound alteration in the quality of life of infertile couples (38).

The consequence of all this social pressure is the use of non-medical practices, such as consulting traditional healers. These beliefs are widely held in Africa in an attempt to explain the cause of their infertility. Over the years, this pressure becomes unbearable, leading infertile women to gradually withdraw into society. They avoid all ceremonies where their lack of children might be the subject of comment. The child represents the descendants of the family. Their absence causes great trauma. This shows the central place that the child occupies in marriage in sub-Saharan Africa, and leads us to understand that the interpretation that African societies in general give to the notion of femininity is inseparable from a woman's ability to procreate.

### 4.6 Limitations

This study allowed us to assess the current state of primary infertility in our practice setting. It provides us with epidemiological, clinical, paraclinical, and psychological data. This study would have been more relevant with a larger sample recruited from several referral centers. Financial support, allowing all included couples to undergo a full infertility assessment, would have further validated this work.

## 5 Conclusion

Infertility remains a major public health problem. Primary infertility is becoming more and more frequent in our context, certainly in relation to maternal age but also to socio-professional category. Infertility is a social tragedy, especially in Africa. The child strengthens the couple's bond even more, so that couples are not exposed to the risk of stigmatisation, marginalisation and pressure from society. The psychosocial impact this can have is enormous. Given this, many patients need psychological support before, during and after treatment; this will enable them to express their experiences and better anticipate possible complications. Medical treatment for infertility should also be included in the health insurance programme to ensure inclusive health for all (39).

## Data Availability

The original contributions presented in the study are included in the article/Supplementary Material, further inquiries can be directed to the corresponding author.
